# The multi-pronged entry of African swine fever virus: from host factors to therapeutic targets

**DOI:** 10.1128/jvi.00924-26

**Published:** 2026-09-23

**Authors:** Jiaqi Li, Chuanxia Liu, Weitao Huang, Yibing Wang, Li Huang, Changjiang Weng

**Affiliations:** 1 State Key Laboratory for Animal Disease Control and Prevention, Harbin Veterinary Research Institute of Chinese Academy of Agricultural Sciences 687216, Harbin, China; 2 Heilongjiang Provincial Key Laboratory of Veterinary Immunology, Harbin, China; Universiteit Gent, Merelbeke, Belgium

**Keywords:** African swine fever virus, viral entry, host factors, endocytosis, membrane fusion, transgenic pig

## Abstract

African swine fever (ASF), caused by the African swine fever virus (ASFV), remains one of the most devastating transboundary animal diseases worldwide, with case fatality rates reaching up to 100% in acute infections. Viral entry represents the first essential step in infection and is an ideal target for the development of vaccines and antiviral drugs. Recent studies have revealed that ASFV entry is not mediated by a single receptor or pathway. Instead, it involves multiple viral structural proteins, cellular attachment factors, endocytic routes, and intracellular trafficking regulators, which form a coordinated virus-host interaction network. In this review, we systematically summarize recent advances in ASFV multi-pathway invasion, including viral attachment, internalization, intracellular trafficking, uncoating, and membrane fusion. Additionally, we discuss several unresolved questions and highlight promising antiviral and genetic intervention strategies that target both viral components and host determinants. A comprehensive understanding of ASFV entry will provide the theoretical foundation for developing next-generation vaccines, antivirals, and disease-resistant pig populations.

## INTRODUCTION

African swine fever (ASF) is a highly lethal hemorrhagic infectious disease that infects both domestic and wild swine, and causes devastating economic losses to the global pig production industry. The causative agent, the African swine fever virus (ASFV), was first isolated in Kenya in the early 20th century (1). Following the introduction of genotype II ASFV into Georgia in 2007, the virus spread throughout Eastern Europe and Asia (2, 3). Since the first outbreak in China in 2018, ASF has remained a major threat to pork production and food security (4). ASFV comprises at least 24 genotypes and exhibits substantial genetic diversity in Africa (5). Importantly, the recent emergence of genotypes I and II, along with their recombinant strains, in Asia has further increased the complexity of ASF epidemiology. This raises concerns about viral evolution, transmission dynamics, and vaccine efficacy (6).

**Fig 1:**
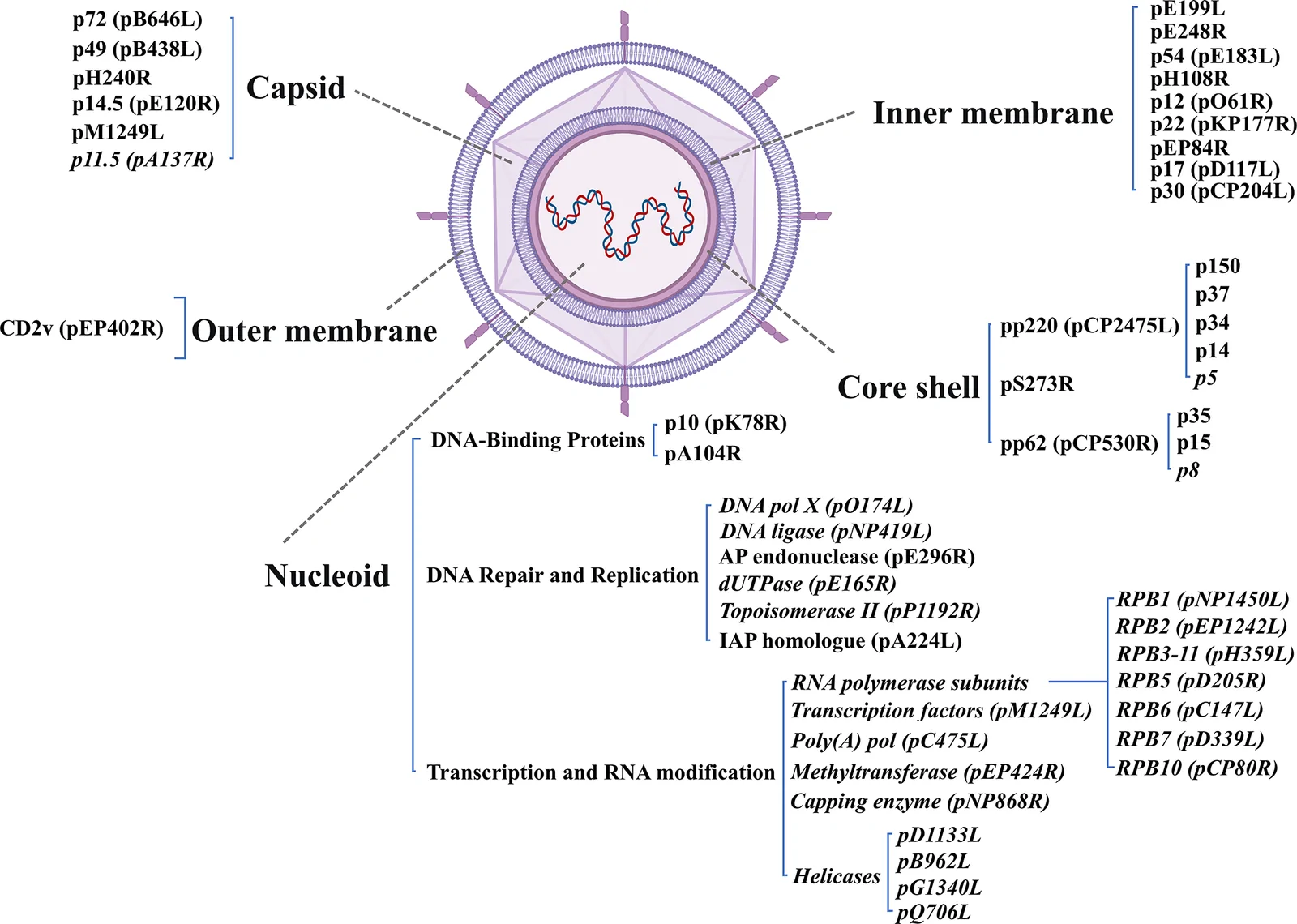
Structure of the ASFV virion. The five-layered structure of the ASFV virion, along with the viral proteins resident in each layer, is depicted in this figure. The localizations of proteins indicated in italics are inferred based on predicted or established functional annotations, while the locations of the remaining proteins are confirmed by experimental evidence. This illustration was created using BioRender.com.

Among all stages of the ASFV life cycle, viral entry into host cells is the earliest and is one of the most vulnerable points for therapeutic and preventive interventions. Successful entry is a prerequisite for viral replication and determines host cell susceptibility. Mature extracellular ASFV particles have a diameter of approximately 200 nm and consist of a five-layer structure from the outside inward: an outer membrane, icosahedral capsid, inner membrane, core shell, and central nucleoid (7). The outer membrane is a lipid bilayer acquired from the host cell membrane during the budding and release stages; it is present only in mature infectious viral particles outside the cell. Its major constituent protein is CD2v (pEP402R) (8). The capsid serves as the core scaffold and is assembled from the major structural protein p72 (pB646L) and four minor structural proteins, pH240R, pM1249L, p17 (pD117L), and p49 (pB438L) (9–11). The inner membrane is a lipid bilayer derived from the host endoplasmic reticulum. Its major constituent proteins include pE199L, pE248R, p54 (pE183L), pH108R, p12 (pO61R), p22 (pKP177L), pEP84R, p17 (pD117L), and p30 (pCP204L) (8, 10, 12, 13). These structural layers perform indispensable functions during viral infection (Fig. 1). Notably, in addition to extracellular virions, intracellular mature virions are also present, analogous to vaccinia virus (VACV) (14). Both forms are infectious (15); however, because intracellular mature virions lack an outer membrane, they likely enter cells via distinct virus- and host-dependent pathways.

Accumulating evidence indicates that ASFV entry is complex and involves multiple viral adhesion proteins, host receptors, and endocytic pathways. In recent years, rapid advances in gene editing technologies, high-resolution microscopy, genome-wide CRISPR screening, quantitative proteomics, and structural biology have significantly expanded our understanding of the ASFV invasion mechanisms. However, several fundamental scientific questions regarding the entry of ASFV invasion remain unanswered. The identities of functional receptors are still incompletely defined, the relative contributions of different internalization pathways remain controversial, and the molecular mechanisms governing viral uncoating and membrane fusion are only beginning to be elucidated.

In this review, we summarize the current knowledge on ASFV entry, including adhesion, internalization, endosomal transport, uncoating, and membrane fusion. We integrated existing virus-host interaction networks, analyzed unresolved scientific questions, and discussed the translational potential of the major targets involved in viral invasion. A comprehensive elucidation of the multi-pathway invasion mechanism of ASFV will advance our knowledge of ASFV strategies and establish a target landscape for next-generation prevention and control strategies.

## ATTACHMENT AND RECEPTOR ENGAGEMENT

Viral attachment is the first step of viral entry into host cells, during which surface proteins on virions bind to molecules on the host cell membrane, which is crucial for initiating viral internalization and represents a promising target for blocking viral entry. It employs a sophisticated strategy involving multiple viral attachment proteins, diverse host attachment factors, candidate receptors, and alternative mechanisms that promote viral entry (Table 1).

**TABLE 1:** Summary of key factors involved in ASFV invasion (viral proteins vs host factors)

Entry stage	Viral protein	Host factors	Functional description	Reference(s)
Attachment	p72	CD1d	Mediating virus binding to the host cell membrane	(16)
	pE120R	RPSA		(17)
	PS on outer membrane/ apoptotic bodies (ApoBD)	Gas6, AXL, and TIM4		(15, 18)
	p72, p17, CD2v, pM1249L (predicted)	HS		(19)
	pA137R	FcγRII/III		(20, 21)
	p12, p54	Not determined		(22–24)
	Not determined	CD163, Siglec1, MYH9, ANXA1		(25–27)
Internalization				
Macropinocytosis	Not determined	EGFR and AXL	Triggering the internalization pathway and signal transduction	(18, 28)
Clathrin-mediated endocytosis (CME)	p72	CD1d, EPS15, AP-2, EPSIN1, and clathrin		(16)
Caveolin-mediated endocytosis (CavME)	pE120R	RPSA and CAV-1		(17)
Endosomal transport and maturation	Not determined	Rab5, Rab7, and TMEM239	Mediating microtubule-dependent endosomal transport and maturation	(29–31)
Uncoating	p72	Stub1, HSPA8, and p62	Low pH and ubiquitination trigger the degradation of p72 to mediate uncoating	(32)
Membrane fusion	pE199L and pE248R	NPC1, NPC2, LAMP1, LAMP2, and WDR91	Fusion of the viral inner membrane with the late endosomal membrane to promote viral genome release	(13, 33–36)

### Viral attachment proteins

Early adhesion-blocking experiments have demonstrated that antibodies against p12, p54 (pE183L), and p72 significantly inhibited the specific binding of viral particles to macrophages, indicating that these structural proteins are important viral adhesion factors mediating ASFV attachment to host cells (22–24). CD2v, a major component of the viral envelope, exhibits erythrocyte-adsorption activity, and binding of CD2v with λ-carrageenan inhibits ASFV adhesion and internalization, further supporting its role as a viral adhesion protein (37). However, the key host cell receptors responsible for viral adhesion at this stage remain unknown.

### Host attachment factors and candidate receptors

Glycosaminoglycans (GAGs) are complex polysaccharides that are widely present on cell surfaces and are considered universal host molecules that various viruses hijack to facilitate their initial adhesion (38). A 2026 study confirmed that heparan sulfate (HS), a major member of the GAG family, is a host factor that mediates the initial non-specific adhesion of ASFV. Pre-incubation of ASFV with soluble HS competitively blocks virus-cell surface HS interaction and inhibits infection; similarly, degrading HS on porcine alveolar macrophages (PAMs) using heparinase I/III also reduces infection efficiency. Pre-treating cells with HS-specific inhibitors significantly impedes ASFV adhesion and internalization. Conversely, the overexpression of exostosin glycosyltransferase 1 (EXT1), a key enzyme in HS biosynthesis, significantly promoted ASFV entry. The ASFV proteins CD2v, p72, p17 (pD117L), and pM1249L directly bind to HS and serve as key adhesion proteins that mediate viral entry (19). Notably, studies on VACV, another large DNA virus with a nucleocapsid, showed that the virus not only completes initial adhesion by binding to HS via the A27L protein (39), but can also interact with chondroitin sulfate, another core member of the GAG family, through the D8 protein to mediate initial viral adhesion (40). However, whether ASFV can utilize other GAG family members on the host cell surface in addition to HS to achieve initial adhesion remains to be investigated.

High-affinity interactions between viral adhesion proteins and specific receptors on the host cell surface constitute the principal molecular basis of ASFV macrophage tropism. Although CD163, a marker of mature macrophage, has been implicated in ASFV infection (41), early flow cytometry analyses indicated that both CD163-positive and -negative cells could be infected, even though markers, such as CD45, CD203a, and CD163, were correlated with infection (25). Moreover, subsequent *in vivo* challenge experiments showed that CD163-knockout pigs exhibited no significant difference in susceptibility to the ASFV Georgia 2007/1 strain compared with wild-type pigs, suggesting that CD163 is not an essential functional receptor for ASFV infection (42). Silencing CD163 or sialic acid-binding immunoglobulin-like lectin 1 (Siglec1, also known as CD169) individually in PAMs did not significantly affect ASFV infectivity; however, the simultaneous silencing of both genes significantly reduced infection efficiency. Furthermore, co-overexpression of CD163 and Siglec1 in non-susceptible PK15 and 3D4/21 cells conferred susceptibility to ASFV, suggesting that CD163 and Siglec1 may act as co-receptors (26). In addition, antibodies targeting Siglec1, myosin heavy chain 9 (MYH9), and annexin A1 (ANXA1) significantly inhibit ASFV entry into PAMs, suggesting that these molecules may serve as potential host receptors or co-receptors for ASFV infection (27). However, the specific roles of these molecules in the ASFV adhesion process and the viral adhesion proteins that interact with them remain unclear. In 2023, a notable study identified CD1d as a functional receptor for ASFV entry. Using IP-MS to screen for host membrane proteins interacting with p72, researchers revealed the molecular mechanism whereby p72 specifically binds to CD1d to mediate viral adhesion. This mechanism recruits the intracellular linker protein EPS15 to initiate clathrin-mediated endocytosis (CME) and ultimately facilitate viral internalization (16). However, further *in vivo* challenge experiments showed that CD1d knockout piglets remained fully susceptible to the highly virulent genotype II ASFV strain MNG19, with no significant differences in disease severity or mortality compared with wild-type pigs, confirming that CD1d is not an essential receptor for viral replication and virulence *in vivo* (43). Because p72, the major structural protein, is not exposed on the outer membrane of extracellular virions (8), the p72-CD1d-dependent entry route likely applies to intracellular virions, whose surfaces expose capsid proteins, and not to extracellular virions. Therefore, CD1d-deficient piglets may still be susceptible to ASFV, as extracellular virions are capable of entering independently of CD1d.

### Alternative attachment strategies

In addition to the use of its own structural proteins as adhesion molecules, ASFV has evolved alternative strategies for attachment and entry. Apoptotic mimicry is a highly conserved entry mechanism whereby viral particles externalize phosphatidylserine (PS) on the surface of the viral envelope, mimicking the “eat me” signal of host apoptotic cells or apoptotic bodies (ApoBD). This mimicry allows the virus to be recognized by host cell PS receptors, thereby facilitating viral attachment and entry (44). ASFV utilizes apoptotic mimicry in two distinct ways. First, free viral particles directly bind to the host tyrosine kinase receptor AXL via PS exposed on the viral envelope, thereby completing viral attachment and subsequently triggering macropinocytosis to mediate viral entry into PAMs. Second, during the late stages of infection, ASFV induces apoptosis in PAMs, resulting in the release of apoptotic bodies that contain single-membrane-enveloped viral particles lacking the outer envelope. Mechanistically, the specific interaction between viral p54 protein and secretory carrier membrane protein 3 (SCAMP3) constitutes a central molecular event governing the formation of virus-laden apoptotic bodies (45). Recently, these apoptotic bodies were shown to be recognized by the phagocytic receptor TIM4 on the surface of PAMs, which binds to PS on the surface of apoptotic bodies, thereby facilitating viral entry via the endocytic pathway and promoting ASFV infection of PAMs (15). Concurrently, ASFV infection significantly upregulates CD14 expression in both infected and bystander cells, enhancing the phagocytic efficiency of PAMs toward ASFV-containing apoptotic bodies and further promoting viral entry and intercellular transmission (18, 46). Furthermore, during viral assembly and budding, viral particles not only acquire PS from the host cell membrane to display it on the envelope surface, thereby mediating viral adhesion and entry, but also specifically recruit and package various host proteins into viral particles to facilitate viral entry into specific cells. For example, HIV-1 incorporates the host ICAM-1 molecule into its envelope, enhancing the virus’s efficiency of adhesion and entry into CD4^+^ T cells (47). Proteomic analyses have indicated that mature ASFV particles contain at least 60 viral-encoded proteins and more than 20 host proteins (8, 9). However, whether ASFV mediates adhesion through host proteins packaged into viral particles, as well as the regulatory role of this mechanism in viral cell tropism, remains to be systematically investigated.

### Antibody-dependent enhancement

Antibody-dependent enhancement (ADE) is a phenomenon in which antibodies not only fail to effectively neutralize a virus but also promote viral entry into immune cells, such as macrophages, by binding to Fcγ receptors (FcγRs) on the cell surface. This has been observed in viruses, including dengue virus, SARS-CoV-2, and Zika virus (48–50). ADE presents a serious challenge in the development of traditional vaccines and antibody therapies. In ASFV, specific antibodies produced by the host against the viral pA137R protein can bind to pA137R on viral particles, forming an antibody-virus complex. PAMs, which highly express FcγRs on their surface (51), recognize and capture these complexes via FcγRII (CD32) and FcγRIII (CD16), thereby facilitating viral adhesion and entry, and ultimately exacerbating ASFV infection. The pA137R has been identified as the principal viral factor responsible for ADE during ASFV infection (20, 21). Knockout of *A137R* may represent a viable approach to mitigate ASFV-associated ADE (52). Alternatively, detailed characterization and engineering of specific functional domains within pA137R that mediate ADE may represent effective approaches to mitigate ADE. Concurrently, the identification of protective ASFV antigens and the subsequent design of subunit or viral-vectored vaccines hold promise for circumventing ADE. Addressing ADE has become a critical scientific challenge and a forefront area of research that demands urgent attention in the context of vaccine and therapeutic antibody development. Successfully overcoming this obstacle will not only eliminate a significant safety concern related to vaccine- and antibody-based interventions but will also substantially advance the understanding of the molecular mechanisms governing ASFV entry into host cells.

## INTERNALIZATION OF ASFV

Following attachment to the host cell surface, ASFV initiates endocytosis by triggering intracellular signaling cascades, thereby facilitating the internalization of viral particles (53). Unlike many viruses that rely predominantly on a single internalization mechanism, ASFV is capable of exploiting multiple endocytic pathways, including macropinocytosis, CME, and caveolin-mediated endocytosis (CavME).

### Macropinocytosis

Macropinocytosis is an actin-dependent, non-selective endocytic process that serves as one of the primary pathways through which various large DNA viruses, such as human cytomegalovirus and vaccinia virus, invade host cells (54–56). During ASFV entry, the virus activates the epidermal growth factor receptor (EGFR), initiating downstream PI3K-Akt and Pak1-Rac1 signaling pathways, which subsequently trigger macropinocytosis. Regulation of Na^+^/H^+^ exchanger activity and actin cytoskeletal reorganization are the major regulatory steps of this pathway (28). Furthermore, ASFV can also utilize apoptotic mimicking to facilitate adhesion by binding to the host receptor AXL via PS externalization, thereby activating the downstream PI3K-Rac1 signaling pathway, driving actin reorganization and membrane invaginations, and ultimately mediating viral entry through macropinocytosis (18).

### Clathrin-mediated endocytosis

In contrast to macropinocytosis, the CME pathway is a classic receptor-mediated endocytic process. Its key feature involves the recruitment and assembly of clathrin at specific sites on the cell membrane to form clathrin-coated pits (CCPs), which subsequently undergo membrane budding and shearing to generate clathrin-coated vesicles (CCVs) that transport viral particles into the cytosolic endosomes (57). For viruses with particle sizes less than 200 nm, such as SARS-CoV-2 and influenza A virus, CME is a common mode of endocytosis (58, 59). In the CME pathway, after ASFV binds to an endocytic receptor, it activates the receptor to recruit endocytic adaptor complexes, such as AP-2, EPS15, and EPSIN1, driving the formation of CCVs to initiate internalization and ultimately delivering viral particles into the cell. For example, it has been reported that upon binding to CD1d, ASFV p72 recruits the intracellular linker protein EPS15 to form a p72-CD1d-EPS15 ternary complex, which subsequently drives clathrin aggregation and CCV formation, revealing the specific molecular mechanism by which ASFV triggers the CME pathway (16). Furthermore, cholesterol is an important regulator of the CME pathway; cholesterol depletion disrupts the structure of lipid rafts in the cell membrane, inhibits the assembly and formation of clathrin-coated vesicles, and consequently significantly blocks ASFV internalization (60).

### Caveola-mediated endocytosis

Although macropinocytosis and CME have long been recognized as major entry pathways for ASFV, a recent study revealed a third pathway: caveola-mediated endocytosis (CavME). Caveolae are flask-shaped invaginations of the plasma membrane that are enriched in cholesterol, sphingolipids, and caveolin proteins. In this pathway, the host ribosomal protein RPSA acts as a binding partner for the ASFV capsid protein pE120R and recruits caveolin-1 (CAV-1) via its 16B domain to form the pE120R-RPSA-CAV1 complex, which initiates CavME and promotes ASFV internalization (17). However, further studies are needed to determine whether CavME serves as a primary entry pathway under physiological conditions or functions as a supplementary pathway in specific cellular contexts.

### Cell-type dependence of ASFV entry pathways

Notably, the primary endocytic pathways relied upon by ASFV during invasion vary among different cell types. ASFV enters Vero cells primarily via CME, rather than via macropinocytosis (61). In PAMs, electron microscopy shows that viral particles are engulfed by macropinocytosis-like membrane protrusions and are also found within clathrin-coated vesicles. Both macropinocytosis and CME-specific inhibitors significantly blocked ASFV internalization. Colocalization analysis further revealed that approximately 68% of viral particles colocalized with the macropinocytic tracer dextran, whereas approximately 25% colocalized with the CME marker transferrin, suggesting that ASFV utilizes both pathways in PAMs, with macropinocytosis serving as the primary route (33, 62). However, most current studies on the internalization mechanism of ASFV have relied on chemical inhibitors, which may cause off-target effects or cytotoxicity. Moreover, the exclusive use of colocalization analyses without corresponding biochemical validation cannot distinguish whether internalized particles undergo productive entry or are instead directed to degradative compartments and subsequently eliminated. Furthermore, given that phagocytosis is a fundamental and essential function of macrophages, ASFV can exploit this pathway to facilitate viral entry into PAMs, for instance, through the phagocytic uptake of infectious intracellular virions (46). These findings collectively indicate that the endocytic mechanism of ASFV is cell-type dependent and that the virus can flexibly employ multiple internalization pathways.

## ENDOSOMAL TRAFFICKING AND MATURATION

Following internalization, ASFV undergoes transport and maturation through the endosomal-lysosomal system before initiating uncoating, membrane fusion, or genomic release. This process is highly dependent on endosomal transport and precise regulation of host factors, representing a current frontier in research on the mechanisms of ASFV infection. Between 5 and 30 min after internalization, ASFV particles were primarily localized in early endosomes and macropinosomes expressing EEA1^+^/TfR^+^/Rab5^+^. During the late phase (30–90 min), the virus is gradually transported via vesicles to late multivesicular endosomes and lysosomes expressing CD63^+^/LAMP1^+^/LAMP2^+^/Rab7^+^/Cathepsin L^+^, thereby laying the groundwork for uncoating and membrane fusion (33). This vesicular transport process is precisely regulated by Rab family small GTPases (63). As a key component of the ASFV inner membrane, p54 directly binds to dynein light chain 8 (DLC8) in the cytoplasm. Since DLC8 is a minus-end-directed microtubule-associated motor protein, the p54-DLC8 interaction may mediate the intracellular transport of virions along microtubules (64). Rab5 and Rab7 are essential molecular switches in endosomal maturation. Rab5 regulates early endosome biogenesis and fusion, whereas Rab7 drives the maturation of early endosomes into late endosomes and their subsequent fusion with lysosomes (30). Silencing Rab7 causes viral particles to accumulate extensively in early endosomes, significantly downregulating the expression of ASFV early protein p30 (pCP204L), thereby blocking infection (31).

Recently, a genome-wide CRISPR-Cas9 screen identified TMEM239 as a host factor that regulates the early vesicular transport of ASFV. Mechanistic studies revealed that TMEM239 directly interacts with Rab5A, a member of the Rab5 family, and promotes ASFV entry into early endosomes by upregulating Rab5A activity. This makes TMEM239 a critical host factor for viral infection. Knockout of TMEM239 significantly reduced ASFV replication efficiency, and peripheral blood mononuclear cells from TMEM239-knockout pigs exhibited significant resistance to ASFV infection. These findings provide novel targets for the development of host-directed antiviral drugs against ASFV and for disease-resistant breeding through gene editing (29).

## UNCOATING, MEMBRANE FUSION, AND GENOME RELEASE

Successful delivery of ASFV particles to the late endosomal and lysosomal compartments does not immediately lead to infection. Before viral gene expression begins, the incoming virion must overcome a series of structural barriers, culminating in a highly coordinated sequence of events, including endosomal acidification, virion disassembly, membrane fusion, and genome release.

### Acidification-triggered uncoating and capsid disassembly

After the virus is transported via early endosomes and macropinosomes to late endosomes containing multiple vesicles and lysosomes, it undergoes a stepwise uncoating process driven by acidic pH: the outer membrane ruptures, followed by disintegration of the capsid (33). Using quantitative electron microscopy, researchers found that rupture of the ASFV outer membrane and disassembly of the viral capsid primarily occur in late endosomes and lysosomes, and that endosomal acidification is a necessary prerequisite for uncoating and membrane fusion. The acidic environment of late endosomes (pH 5.0–6.0) activates endosomal proteases and triggers conformational changes in viral proteins, creating conditions for capsid disassembly and membrane fusion. Inhibition of endosomal acidification using the H^+^-ATPase inhibitor Bafilomycin A1 causes viral particles to become trapped in Rab7^+^ late endosomes, preventing the completion of uncoating and membrane fusion, thereby completely halting the ASFV infection process (34, 65, 66).

Capsid disassembly is an important step in the uncoating of ASFV. Recently, a study first revealed an autophagy-mediated mechanism in which the capsid protein p72 is ubiquitinated by the E3 ubiquitin ligase Stub1. The ubiquitinated p72 then binds to the autophagy receptor p62, a process mediated by the chaperone HSPA8, and is transported into an autophagosome. p72 degradation and capsid disassembly are ultimately completed within the autolysosome. Inhibition of the autophagy pathway or knockdown of Stub1, HSPA8, or p62 expression significantly inhibits ASFV uncoating and subsequent infection, filling a gap in our understanding of the mechanisms in this field (32). In addition, although the C-terminal domain of p72 is required for its ubiquitination, the precise sites are undefined (32). Since endosomal acidity promotes conformational rearrangements of viral proteins (e.g., influenza HA) (67), we propose that it may likewise alter p72 conformation, exposing its ubiquitination sites. This exposure would then trigger ubiquitination and autophagy-mediated degradation, thereby promoting uncoating.

Studies on VACV have demonstrated that its uncoating process depends not only on acidic pH and ubiquitination but also critically on early viral gene expression, particularly D5 protein. VACV utilizes newly synthesized D5 to interact with the viral core, thereby facilitating the degradation of the outer protein coat surrounding the core and ultimately enabling genome release (68). Evidence indicates that ASFV initiates early transcription as early as 0.5 h post-internalization, producing early proteins, such as p30 (69). Therefore, ASFV may similarly rely on the functions of its early transcription genes during entry, although the precise mechanisms remain to be fully elucidated.

### Viral-endosomal membrane fusion

**Fig 2:**
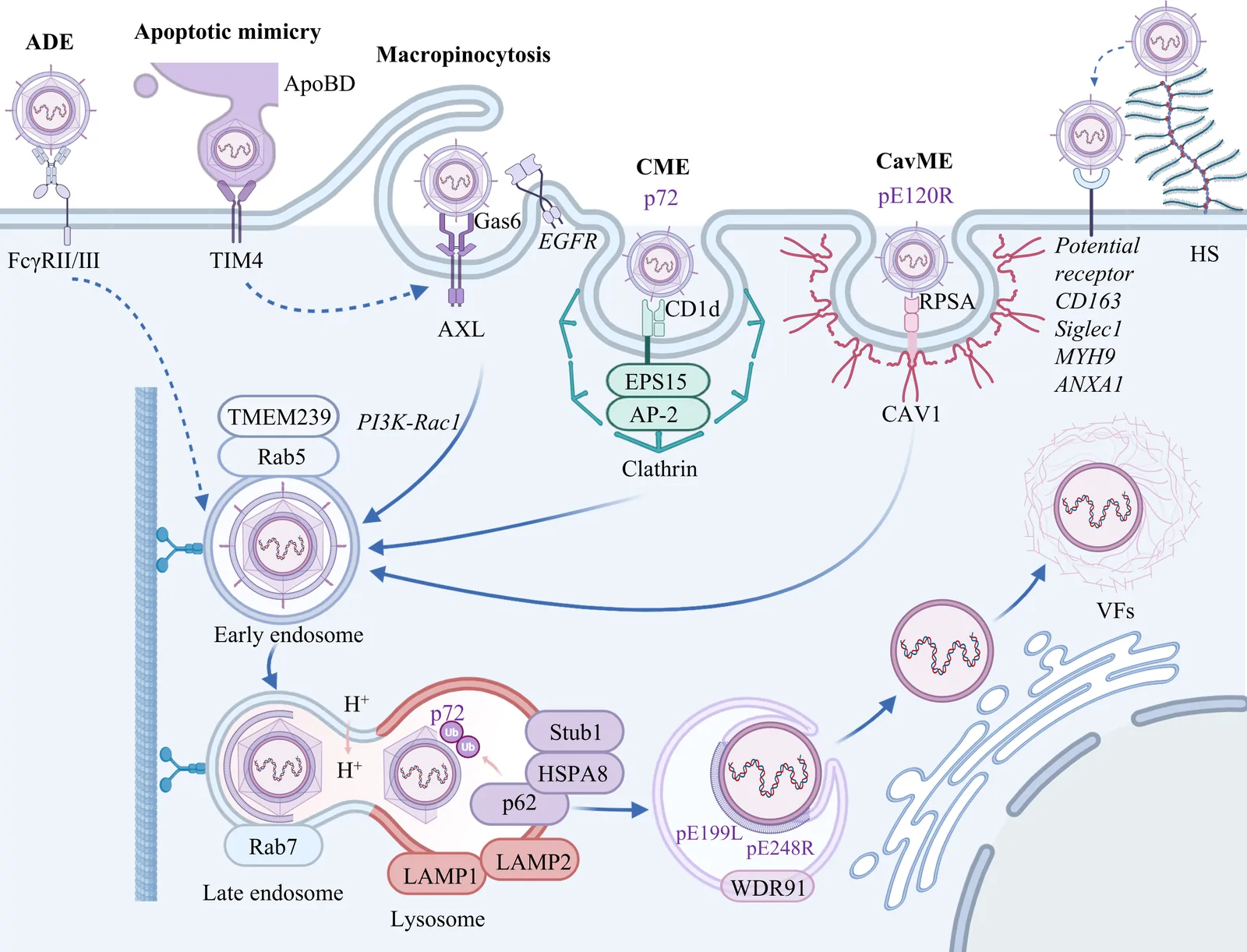
Mechanism of ASFV entry into host cells. ASFV entry into porcine alveolar macrophages (PAMs) involves complex pathways, with candidate or putative mechanisms and host factors indicated by dashed arrows and italic fonts, respectively. Entry is initiated by the attachment of the virus (CN/GS/2018 strain) to cell surface heparan sulfate (HS), followed by engagement with an unknown master receptor that triggers internalization. Subsequently, the virus (HLJ/18 strain) enters early endosomes via macropinocytosis, clathrin-mediated endocytosis, and caveola-mediated endocytosis. It then traffics through and matures within the endo-lysosomal system, ultimately undergoing uncoating, membrane fusion, and genome release to complete the entry process. Additionally, apoptotic mimicry (observed in HLJ/18 and CADC_HN09 strains) and antibody-dependent enhancement (observed in HLJ/18 and JS/LG/21 strains) represent important mechanisms for both viral entry and cell-to-cell spread. This figure was created using BioRender.com.

Following capsid lysis, the inner envelope of ASFV is exposed, and its fusion with the late endosomal membrane constitutes the final step in the viral genome release. The inner envelope proteins pE199L and pE248R are key molecules that mediate membrane fusion; both contain typical fusion peptide domains. Viral particles lacking either of these proteins can adhere to and internalize within the host cells, completing outer membrane shedding and capsid degradation. However, they are unable to complete membrane fusion and genome release, resulting in loss of infectivity. This suggests that pE199L and pE248R act synergistically to mediate membrane fusion (13, 33–35). A recent study demonstrated that the host protein WDR91 specifically interacts with pE248R and pE199L during the viral membrane fusion step, facilitating fusion between the viral inner envelope and endosomal membrane, thereby facilitating the release of the viral core into the cytosol (36). Furthermore, pE199L binds to endosomal proteins NPC1, LAMP1, and LAMP2, whereas pE248R binds to NPC1 and LAMP2. Knocking out NPC1 or inhibiting its function significantly inhibits viral infection, where NPC2 acts in concert with NPC1 to regulate late-stage endosomal cholesterol efflux, suggesting that the ASFV membrane fusion process may depend on the synergistic action of NPC1 and NPC2 to regulate cholesterol flux (34). However, the precise molecular mechanism by which pE199L and pE248R utilize host cellular factors to achieve membrane fusion remains unclear. The known invasion process of ASFV is summarized in Fig. 2.

### Genome release

After the release of ASFV genomic DNA, it predominantly localizes to the perinuclear cytoplasm where it forms viral factories (VFs). These VFs serve as sites for viral genome replication, protein synthesis, and virion assembly (70). The single-round replication cycle of ASFV lasts approximately 9 h: early mRNA transcription begins as early as 0.5 h post-infection (hpi), DNA replication is initiated at 4–5 hpi, viral morphogenesis starts at 6 hpi, and mature virions appear from 9 h onward (69).

## OUTSTANDING ISSUES AND FUTURE DIRECTIONS

Despite significant advances in understanding ASFV invasion mechanisms, this field still faces several critical scientific questions that urgently need to be addressed.

### Is there a master receptor for ASFV?

Substantial gaps remain in our understanding of the modes of interaction between viral proteins and host receptors. ASFV exhibits extensive genotypic diversity with at least 24 genotypes identified to date. Whole-genome sequence similarity among isolates from different genotypes ranges from 90.59% to 99.95% (71, 72). Entry-related proteins, such as p72 and CD2v, display high variability across genotypes (73, 74). However, whether the entry mechanisms differ or share commonalities among these genotypes has not yet been systematically elucidated. It also remains unclear whether variations in the genes encoding adhesion proteins alter their interaction modes and affinities with host receptors, thereby modulating viral cell tropism and pathogenicity. CD2v is a critical virulence factor, and its sequence variation or deletion directly affects virulence and host tropism; however, whether this attenuation results from altered adhesion function requires further confirmation (75, 76).

### Do different ASFV genotypes use different entry strategies?

Existing studies have largely focused on genotype II strains (e.g., HLJ/18, Georgia 2007/1), whereas research on the entry mechanisms of genotype I and genotype I/II recombinant strains remains extremely limited. Notably, genotype I/II recombinant strains that have emerged in recent years in countries such as China and Vietnam exhibit faster evolutionary rates, increased transmissibility, and higher lethality. Moreover, these strains can overcome the immune protection conferred by genotype II-based gene-deleted vaccines (77, 78). This underscores the urgent need to systematically investigate differences in entry mechanisms among distinct ASFV genotypes.

### How does cellular adaptation remodel viral entry?

The remodeling of viral entry mechanisms is a critical step in the acquisition of cellular adaptation. For example, the virulent strain of measles virus primarily relies on the binding of the hemagglutinin (H) protein to the signaling lymphocyte activation molecule (SLAM) receptor to mediate entry, whereas the attenuated strain adapts to Vero cells by utilizing the CD46 receptor (79). Among enteroviruses, the attenuated strain exhibits markedly enhanced affinity for the non-specific receptor HS and reduced dependence on pH for viral uncoating compared to the virulent strain, thereby altering its cellular adaptation (80). Studies on the cellular adaptation of ASFV have shown that the virus acquires adaptive changes mainly through gene deletions or serial passages in non-permissive cells. The prevailing view holds that this process is primarily driven by deletions or mutations in the multigene family (MGF) genes. For instance, after serial passage in CA-CAS-01-A cells, the ASFV-MEC-01 strain acquired a deletion of 12 MGF genes in the left variable region of its genome (81), and serial passage of the HLJ/18 strain in HEK293T cells led to mutations in multiple MGF300 and MGF360 genes (82). The ASFV-G/VP110 strain, after 110 passages in Vero cells, also lost several MGF genes and concomitantly gained cellular adaptation (83).

However, while extensive deletions or mutations occur in MGF family genes, the viral genome frequently harbors accompanying point mutations in other critical genes, such as E199L, a key gene involved in membrane fusion (83). This suggests that beyond the MGF family, whether mutations in other key ASFV entry proteins exist and whether these mutations mediate the acquisition of cellular adaptation by remodeling the entry process remain scientific questions requiring further elucidation. Systematically dissecting the similarities and differences in the entry mechanisms of different ASFV genotypes and identifying entry protein mutations beyond MGF that drive changes in cellular adaptation are of great significance for a comprehensive understanding of the pathogenesis and adaptation mechanisms of ASFV.

### What remains unknown about uncoating and membrane fusion?

The signaling cascade mechanisms underlying the interaction between core functional receptors and the initiation of endocytosis remain unclear. It remains unknown whether the putative cellular receptors involved in ASFV entry trigger endocytic signals and the underlying molecular mechanisms. Furthermore, different strains exhibit markedly different dependencies on the endocytic pathway across various cell types, suggesting that multiple functional receptors or co-receptors may act in concert to mediate viral adhesion and internalization. The processes involved in viral uncoating, membrane fusion, and subsequent core release remain largely unknown. Although some studies have examined the role of p72 in uncoating (32), there has been virtually no research on outer membrane shedding and its molecular mechanisms. Regarding the membrane fusion stage, how pE199L and pE248R utilize the endosomal proteins NPC1 and NPC2 to facilitate membrane fusion and core release requires further investigation. The unresolved nature of these issues severely limits our understanding of the ASFV invasion process and hinders the development of antiviral strategies targeting this stage.

### Does the morphological abnormality caused by the deletion of ASFV structural proteins necessarily impair viral entry function?

Notably, during the assembly of ASFV particles, deletion of several genes encoding structural proteins leads to pronounced abnormalities in viral morphology. Specifically, the deletion of the minor capsid proteins at pH240R (84) and pB438L (85), or the inner membrane protein at pH108R (86), severely disrupts the virus assembly process, resulting in the accumulation of abundant aberrant tubular and bilobed virus particles within VFs. This in turn causes a drastic reduction in the yield of infectious mature virions and severely impairs viral infectivity. Abnormal viral morphology may be accompanied by defects in viral attachment and internalization. This phenomenon has also been demonstrated in enterovirus A71 (EV-A71), where point mutations in the VP1 protein disrupt capsid assembly, yielding a large number of morphologically irregular virions and a significantly reduced proportion of mature particles. Concurrently, the mutant exhibits decreased binding to the primary receptor SCARB2, ultimately impairing viral attachment and internalization efficiency (87). However, in ASFV, although deletion of pH240R, pB438L, and pH108R induced similar morphological abnormalities, existing studies indicate that virions produced by the pH240R-deleted strain showed no statistically significant difference in attachment and internalization compared with those of the wild-type strain. These results suggest that pH240R is not an essential protein for ASFV entry into host cells and that its deletion does not affect the proper assembly or function of viral surface entry-related proteins. Whether the reduced infectivity caused by the deletion of pB438L and pH108R is attributable to impaired viral entry capacity currently lacks direct experimental evidence, which remains to be elucidated through further studies.

### Beyond classical entry: alternative routes of viral dissemination

The relative contributions and regulatory roles of various adhesion pathways, including classic viral-host cytokine interactions, apoptotic mimicry, and ADE, in ASFV infection remain unclear, particularly because of the lack of systematic validation and quantitative assessment under physiological *in vivo* conditions. Therefore, identifying the core functional receptors involved will be a critical direction in the prevention and control of ASFV infection.

Increasing evidence from diverse viral systems suggests that the infection is not limited to classical cell-free transmission. Alternative mechanisms include migrasomes formed at the ends of retraction fibers during cell migration (88), exosomes released by the fusion of multivesicular bodies with the plasma membrane in the endosomal pathway (89), tunneling nanotubes (TNTs) that connect the cytoplasm of adjacent cells, and apoptotic body-mediated intercellular transfer (15, 45). Among them, TNTs have been reported to potentially participate in the intercellular transmission of ASFV (90), and apoptotic bodies containing ASFV particles have been demonstrated to mediate efficient cell-to-cell spread via PS-TIM4 recognition (15). However, to date, no studies have reported the roles of migrasomes and exosomes in ASFV infection. Migrasome formation is strictly dependent on cell migration and predominantly occurs in cells with strong migratory capacity (91), such as monocyte-macrophages, which are the primary target cells of ASFV infection. In contrast, exosomes are constitutively secreted vesicles produced by nearly all nucleated cells, with immune cells being one of the major contributors (92). Based on these cellular biological characteristics, it is highly likely that ASFV hijacks migrasomes and exosomes to enhance viral entry and spread efficiency.

## THERAPEUTIC OPPORTUNITIES EMERGING FROM THE BIOLOGICAL CHARACTERISTICS OF ASFV ENTRY

Antiviral strategies targeting the viral invasion process can disrupt the viral life cycle at the earliest stages of infection, thereby preventing viral replication and immune damage caused by the viral genome upon entry into host cells. This approach represents one of the most promising approaches for the development of anti-ASFV drugs. Based on the analysis of the invasion mechanism presented in this paper, several highly promising targets have been identified, which can be categorized into two main groups: viral and host targets.

### Viral targets

Regarding viral targets, p72, p54, and CD2v, which mediate viral adhesion, and pE199L and pE248R, which mediate membrane fusion, are the priority targets with the greatest potential for development. Developing peptide inhibitors or neutralizing antibodies that target the conserved domains, where p72, p54, and CD2v interact with cellular receptors, holds significant promise (93, 94). For instance, the aforementioned λ-carrageenan has been demonstrated to interfere with viral attachment and internalization processes through its interaction with CD2v (37). In addition, gene editing to mutate the interaction sites between these viral proteins and their receptors, or to delete genes involved in infection to create replication-deficient vaccines and complementary cell lines, is another important direction in vaccine research and development. For example, a monkeypox virus replication-deficient vaccine lacking the OPG96 and OPG158 genes illustrates this approach (95).

### Host targets

Regarding host-targeted molecules, HS mediates initial adhesion, CD1d facilitates specific recognition, AXL initiates macropinocytosis, Rab5/Rab7 and TMEM239 regulate endosomal transport, and the key components of the Stub1/HSPA8/p62 autophagy pathway, which mediate capsid disassembly. All these represent potential host-targeted drug targets. For example, cepharanthine, an inhibitor that targets CME, can block the endocytosis of ASFV, thereby inhibiting viral infection (18, 93, 95). In addition, it inhibits viral invasion through a dual mechanism: on one hand, it disrupts the interaction between profilin 1 and actin to suppress actin remodeling; on the other hand, it reduces cellular phosphatidylethanolamine levels to decrease cell membrane permeability (96).

Compared with virus-targeted drugs, host-targeted therapies can effectively circumvent drug resistance caused by viral genetic mutations and may exhibit broad-spectrum inhibitory activity against multiple genotypes of ASFV. Identifying the cellular receptors essential for ASFV invasion and employing gene-editing technologies to develop ASF-resistant pig breeds is a critical strategy for preventing and controlling ASF at its source. Traditional ASF prevention and control primarily rely on biosecurity measures and stamping-out policies, whereas disease resistance breeding, by modifying host cell genes required for viral infection, endows pigs with natural resistance to ASFV and holds the potential to fundamentally resolve the ASF epidemic.
